# Automatic Extraction of Tunnel Lining Cross-Sections from Terrestrial Laser Scanning Point Clouds

**DOI:** 10.3390/s16101648

**Published:** 2016-10-06

**Authors:** Yun-Jian Cheng, Wenge Qiu, Jin Lei

**Affiliations:** MOE Key Laboratory of Transportation Tunnel Engineering, Southwest Jiaotong University, Chengdu 610031, China; by_sky@my.swjtu.edu.cn (Y.J.C.); leijin14@my.swjtu.edu.cn (J.L.)

**Keywords:** cross-section, filtering, terrestrial laser scanning, tunnel, point cloud

## Abstract

Tunnel lining (bare-lining) cross-sections play an important role in analyzing deformations of tunnel linings. The goal of this paper is to develop an automatic method for extracting bare-lining cross-sections from terrestrial laser scanning (TLS) point clouds. First, the combination of a 2D projection strategy and angle criterion is used for tunnel boundary point detection, from which we estimate the two boundary lines in the X-Y plane. The initial direction of the cross-sectional plane is defined to be orthogonal to one of the two boundary lines. In order to compute the final cross-sectional plane, the direction is adjusted twice with the total least squares method and Rodrigues’ rotation formula, respectively. The projection of nearby points is made onto the adjusted plane to generate tunnel cross-sections. Finally, we present a filtering algorithm (similar to the idea of the morphological erosion) to remove the non-lining points in the cross-section. The proposed method was implemented on railway tunnel data collected in Sichuan, China. Compared with an existing method of cross-sectional extraction, the proposed method can offer high accuracy and more reliable cross-sectional modeling. We also evaluated Type I and Type II errors of the proposed filter, at the same time, which gave suggestions on the parameter selection of the filter.

## 1. Introduction

Over the past years, terrestrial laser scanning (TLS) has been used in inverse engineering [1,2,3], for tracking the changes of natural surfaces via the comparison of different point clouds [4,5,6,7,8] and in the estimation of forest attributes [9,10,11]. The use of the TLS technique has also become popular in tunnel engineering due to the various advantages over conventional geodetic devices, such as laser beam profilers and total stations, which take more time to acquire data [12] and cannot offer high- density 3D datasets. The existing applications of TLS for tunnels contain geological feature detection [13], deformation analysis [14,15,16], and cross-sectional extraction [12,17], and the automatic processing of tunnel point clouds has received increasing research attention [12,18].

The checking of tunnel cross-sections is the primary method for deformation monitoring and clearance inspection. In addition to conventional geodetic surveys, several methods have been developed to extract tunnel cross-sections based on digital photogrammetry or the TLS technique. Combining photogrammetry and laser-lit spots, Wang et al. [19] improved a profile-image method for measuring cross-sections. Their method overcomes the limit of the number of points that conventional geodetic surveying has, but it is difficult to provide sufficient lighting conditions in an actual tunnel. TLS can offer active measurement even in a tunnel where there is no light, which makes it a preferred technique for the extraction of tunnel cross-sections. Several scholars used standard geometric models, such as an ellipse [15] or a circular cylinder [14], to approximate tunnel point clouds, and then estimated cross-sections from the fitted models. However, the non-lining points (i.e., pipes and equipment attached to the lining) must be filtered out for a best fit before approximation, which is difficult from disordered point clouds. Han et al. [12] used a vertical cross-sectional plane to generate tunnel cross-sections. In this approach, they used the surrounding point clouds to determine the directions of cross-sectional planes, and then tunnel cross-sections were generated by projecting the nearby points onto these planes. Based on Han’s method [12], Kang et al. [17] used the quadric-parametric-surface interpolation to compute cross-sectional points where point clouds remained discrete so that they could continuously extract cross-sections.

The so-called bare-lining cross-sections only represent the geometric shapes of tunnel linings. Since non-lining points have no contribution on the coverage of the monitored or inspected cross-section, they are manually or automatically removed in many studies. To ensure the highest filtering quality, Van Gosliga et al. [14] and Nuttens et al. [20] manually removed the points clearly not belonging to the cylindrical linings of the tunnel; nevertheless, manual removal is not economically feasible for a long tunnel and it is very time consuming. Kang et al. [17] used circle fitting to reduce non-lining points of extracted cross-sections, but their method can only be used in circular tunnels. Yoon et al. [18] developed a locomotive-type tunnel scanner using near-infrared laser pulses, based on which they proposed an automated algorithm to extract installations on tunnel linings using the geometric and radiometric features. However, their method cannot work well in a stationary scanning system because the intensity values of tunnel linings provided by TLS vary widely—the intensity varies inversely with the square of the distance between the sensor and the target [21]. To overcome the difficulty of automatically filtering out non-lining points from non-circular lining cross-sections, we designed an angle-based filter to remove non-lining points using a different strategy that is closely related to morphological filter.

The aim of this paper is to propose an automatic method for extracting tunnel cross-sections from TLS point clouds and removing non-lining points of cross-sections. Compared with previous studies, the presented method has two advantages, as follows: (1) the influence of the tunnel grade on the extraction precision of cross-sectional points is considered; and (2) a filtering algorithm for automatically removing non-lining points from arbitrarily-shaped lining cross-sections is proposed. This paper is organized as follows: the method, including estimation of tunnel boundary lines and extraction of bare-lining cross-sections, is introduced in Section 2; Section 3 discusses the experimental results; and, finally, the conclusion is summarized in Section 4.

## 2. Methods

**Figure 1:**
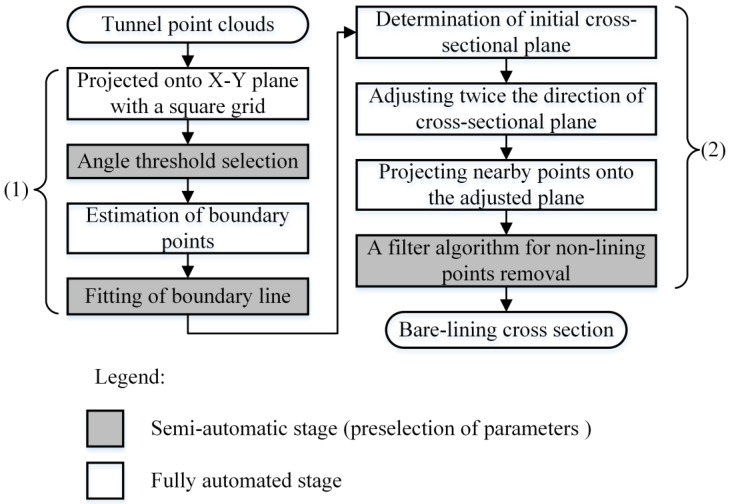
Flowchart of the proposed method.

As shown in Figure 1, the method proposed in this paper is structured in two phases: (1) estimation of tunnel boundary lines; and (2) extraction of bare-lining cross-sections. In the first phase, tunnel boundary points in the X-Y plane are estimated using a 2D projection strategy and angle criterion, and then these boundary points are smoothed to extract boundary lines using polynomial approximation and the RANSAC (RANdom Sample Consensus) algorithm. In the second phase, the initial direction of the cross-sectional plane is defined to be orthogonal to the estimated boundary line, and its direction is adjusted twice to determine the final cross-sectional plane. After projecting the subset of the raw tunnel point clouds onto the final cross-sectional plane, the bare-lining cross-section is extracted using a filtering algorithm. Except the parameter selections, the proposed framework automatically implements the extraction of bare-lining cross-sections.

### 2.1. Estimation of the Tunnel Boundary Lines in the X-Y Plane

An entire tunnel is generally a long tube, so it can be scanned along its centerline station by station. The tunnel point clouds are registered in a user-specified coordinate system using sphere reference targets. In this system, the origin is near the tunnel entrance, and the Y axis is oriented along the direction of the initial segment of the tunnel. Several algorithms [22,23,24] have been developed to quickly extract features from LIDAR point clouds based on projection and gridding. Projecting the scanned data onto the X-Y plane can simplify the 3D tube to a long and narrow 2D object, from which the two boundary points’ groups are extracted from the both sides of the 2D object. In order to improve the speed of extraction, an algorithm for extracting the boundary point groups is proposed using a fixed grid.

**Figure 2:**
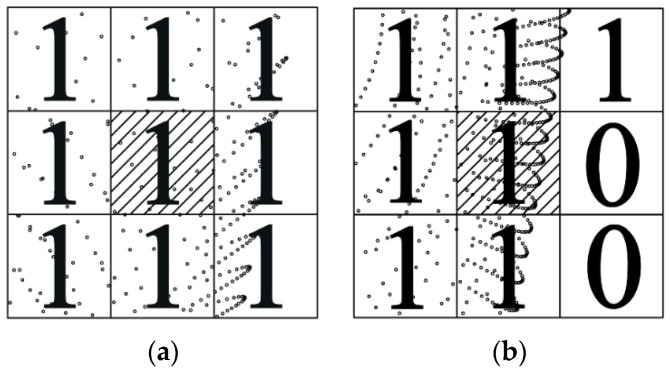
The criterion for determining whether a cell is a boundary or not. (**a**) Non-boundary; and (**b**) boundary.

The projections of the entire tunnel point clouds in the X-Y plane are discretized using a square grid. A grid size that is too large or too small will decrease the computational efficiency or the extraction precision of boundary cells, respectively. The appropriate size of the grid is about one-twentieth of the width of the tunnel. The value of $N_{ij}$ is used to determine whether points exist or not in the cell ij, $N_{ij}$ has a value of 1 if points exist and 0 if there are no points. The empty cell ($N_{ij}$ = 0) is obviously a non-boundary cell; in Figure 2, a 9 × 9 sub-gird consisting of the cell ij and eight neighboring cells is used to determine if the cell ij is a boundary cell or not when $N_{ij}$ = 1, which is formulated as:

(1) $$F_{ij}=\overset{i+1}{\underset{m=i-1}{\prod }}\overset{j+1}{\underset{n=j-1}{\prod }}N_{mn}\;\left\{\begin{array}{c} F_{ij}=1:\text{non-boundary cell}\\ F_{ij}=0:\text{boundary cell} \end{array}\right\}$$

**Figure 3:**
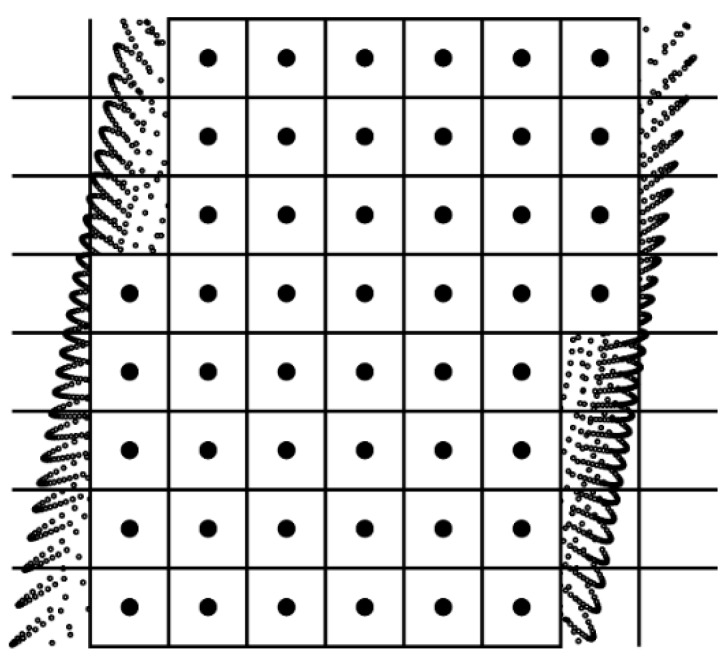
The simplified point clouds used for the further extraction of boundary points.

The center points of non-boundary cells will be used instead of all points in them for the further extraction of boundary points (Figure 3).

**Figure 4:**
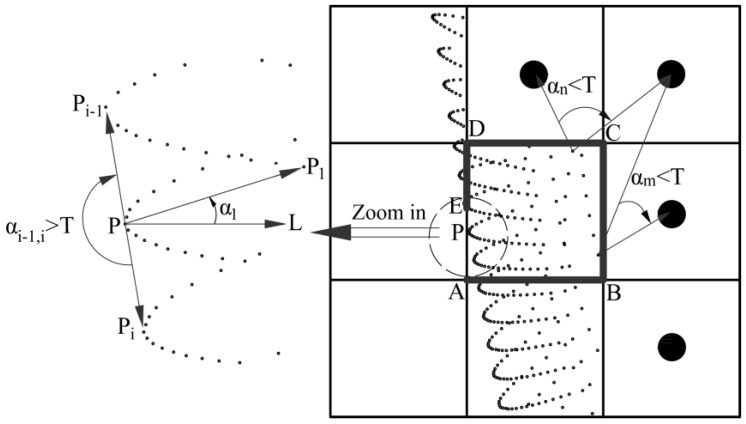
Extracting boundary points from the 9 × 9 sub-girds.

As shown in Figure 4, the point of interest P is an arbitrary point in a boundary cell. We use its eight neighbor cells for avoiding the incorrect extraction of the pseudo-boundary points near the bounding rectangle ABCDE. An angle criterion is proposed based on the distribution of neighboring points (the center points of non-boundary cells and all points in the boundary cells) of point P in the 9 × 9 sub-gird. Cartesian coordinates of the neighbor points are converted to polar coordinates, with the pole set at point P and polar axis L oriented along the positive direction of the X axis. The angular coordinates (e.g., $\alpha_{1}$) of all neighboring points are sorted by value, and then the differences (e.g., $\Delta \alpha_{i-1,i}$) between two consecutive neighboring coordinates are computed. Point P is a boundary points, if the maximum difference exceeds a pre-specified threshold (T), and a non-boundary point, otherwise. This angle threshold (T) is set to 175°, and can work well even in a curved tunnel, because the radius of curvature of the curve segment is large enough (generally greater than 200 m) to consider that the curved boundary in the area of 9 × 9 sub-gird is nearly straight.

Since the tunnel boundary line can be used to determine the initial directions of cross-sectional planes, a cubic polynomial function is chosen to smooth and represent tunnel boundary points, which is parameterized as follows:

(2) $$y=a_{b}x^{3}+b_{b}x^{2}+c_{b}x+d_{b}$$

where $a_{b}$, $b_{b}$, $c_{b}$, and $d_{b}$ are the parameters of a boundary line.

The points belonging to the measurement errors or different structures (i.e., boundary points of refuge recesses) will also be extracted if they meet the angle criterion. The RANSAC algorithm was first proposed by Fischler and Bolles [25] and it is an iterative method to estimate parameters of a mathematical model from a set of observed data which contains outliers. Hence, we adopt this algorithm to find the real boundary points and estimate the parameters of Equation (2).

### 2.2. Extraction of Bare-Lining Cross-Sections

#### 2.2.1. Extraction of Cross-Sections

**Figure 5:**
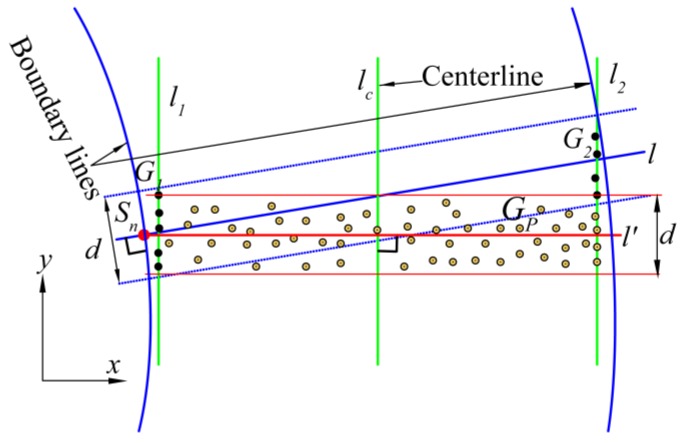
The first adjustment for the direction of cross-sectional plane.

**Figure 6:**
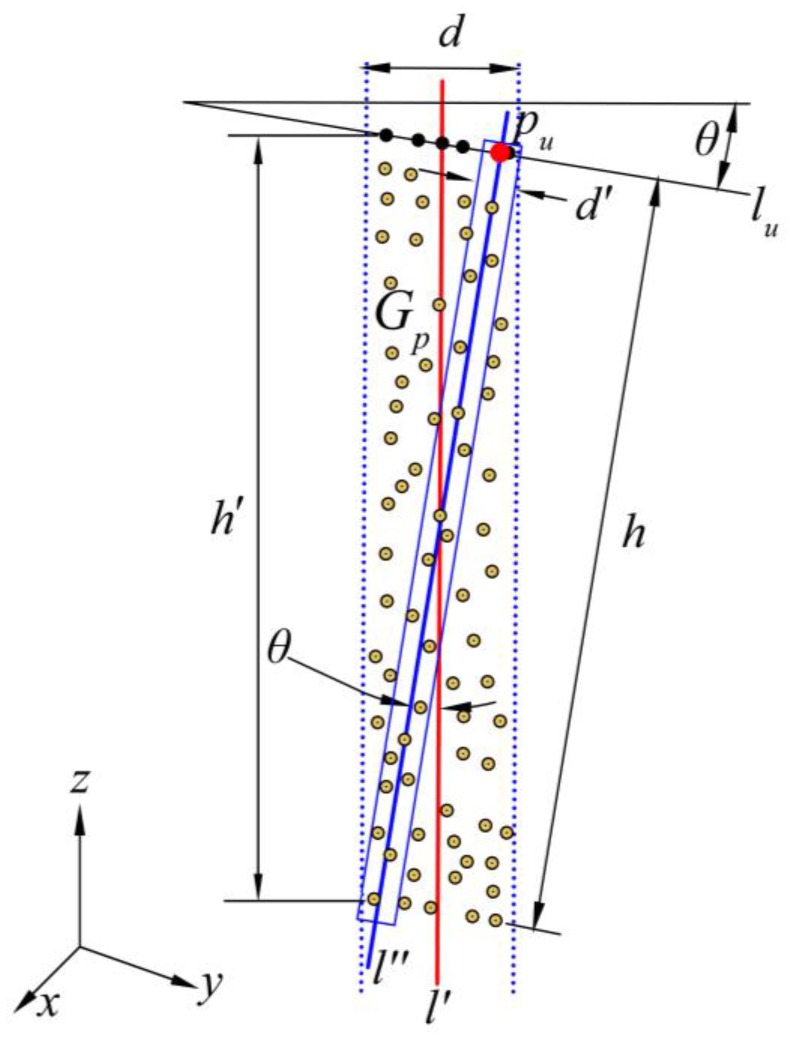
The second adjustment for the direction of cross-sectional plane (a view of the vertical plane traversing centerline $l_{c}$).

After estimation of the two tunnel boundary lines, we define the initial direction of cross-sectional planes to be orthogonal to one of the two boundary lines. Due to the construction and measurement errors and rough concrete linings, even a fine estimation of tunnel boundary lines cannot ensure that the plane orthogonal to it is the real cross-sectional plane. In order to find the real cross-sectional plane, we make adjustments twice to the direction of the initial cross-sectional plane based on the estimations of the local centerline (line $l_{c}$ in Figure 5) and upper boundary line (line $l_{u}$ in Figure 6).

As shown in Figure 5, point $S_{n}\left(x_{S_{n}},y_{S_{n}}\right)$ is selected along one boundary line that was estimated in Section 2.1, depending on the location of the cross-section of interest. The initial cross-sectional plane l is a vertical plane determined from point $S_{n}\left(x_{S_{n}},y_{S_{n}}\right)$ orthogonal to the boundary line. In the first adjustment, the subsets $G_{1}$ and $G_{2}$ (black points) of the two boundary point groups in the X-Y plane are extracted from between the two planes that are parallel to plane l at a distance d. As illustrated in Section 2.1, the two boundary lines of the tunnel can be considered as two straight lines on a small scale, so the value of d should be small, but at the same time it must be large enough to provide enough data for the estimation of the centerline $l_{c}$ (the intersection line of the real cross-sectional plane and X-Y plane should be orthogonal to this centerline). We propose an algorithm to directly estimate the centerline $l_{c}$ from the subsets $G_{1}$ and $G_{2}$ with the total least squares method [26]. The least squares and total least squares methods assess the fitting accuracy in different ways [27]: the least squares method minimizes the sum of the squared vertical distances from the data points to the fitting line, while the total least squares method minimizes the sum of the squared orthogonal distances from the data points to the fitting line.

Since two local boundary lines $l_{1}$ and $l_{2}$ are parallel and have the same distance from the centerline $l_{c}$, the boundary lines are formulated as:

(3) y=ax+b±c

where a and b are the parameters of centerline $l_{c}$, and c is the parameter of boundary lines $l_{1}$ and $l_{2}$.

By using subsets $G_{1}$ and $G_{2}$, the constraint equation for fitting the local centerline $l_{c}$ is derived from Equation (3):

(4) BX=L

where:

(5) $$B=[\begin{array}{ccc} x_{1,1} & 1 & 1\\ \vdots & \vdots & \vdots \\ x_{1,n_{1}} & 1 & 1\\ x_{2,1} & 1 & -1\\ \vdots & \vdots & \vdots \\ x_{2,n_{2}} & 1 & -1 \end{array}],X=\left[\begin{array}{c} a\\ b\\ c \end{array}\right],L=\left[\begin{array}{c} y_{1,1}\\ \vdots \\ y_{1,n_{1}}\\ y_{2,1}\\ \vdots \\ y_{2,n_{2}} \end{array}\right]$$

where $n_{1}$ and $n_{2}$, respectively, denote the numbers of points in subsets $G_{1}$ and $G_{2}$.

We define $U\sum V^{T}$ to be the singular value decomposition of the augmented matrix [B L], where $\sum =\mathrm{diag}\left(\sigma_{1},\sigma_{2},\sigma_{3},\sigma_{4}\right)$ and $\sigma_{1}>\sigma_{2}>\sigma_{3}>\sigma_{4}$. Since B is a full rank matrix (all points in subsets $G_{1}$ and $G_{2}$ are different), the total least squares approximate solution $\hat{X}$ for X is given by:

(6) $$\hat{X}={\left(B^{T}B-\sigma_{4}^{2}I_{3}\right)}^{-1}B^{T}L$$

After the estimation of the centerline $l_{c}$, plane l is adjusted to be plane $l^{\prime }$ whose direction from point $S_{n}\left(x_{S_{n}},y_{S_{n}}\right)$ is orthogonal to centerline $l_{c}$, so the cross-sectional plane $l^{\prime }$ is formulated as:

(7) $$y=-\frac{x}{a}+y_{S_{n}}+\frac{1}{a}x_{S_{n}}$$

In the second adjustment, a point group $G_{p}$ is extracted from between the two planes that are parallel to plane $l^{\prime }$ at a distance d (see Figure 5), and then projected onto the vertical plane traversing centerline $l_{c}$ (see Figure 6). The upper boundary points are extracted using the method proposed in Section 2.1. To find the angle θ of the rotation, the upper boundary line $l_{u}$ is fitted by using those boundary points with the total least squares method.

Vector v is the normal vector of plane $l^{\prime }$, and vector u is the unit vector whose direction from point $S_{n}$ is along the projection of plane $l^{\prime }$ onto the X-Y plane. According to Rodrigues’ rotation formula [28], vector v is rotated by an angle of θ about the axis in the direction of u, from which we can obtain the normal vector $v^{\prime }$ of plane $l^{\prime \prime }$:

(8) $$v^{\prime }=\left(v_{x}^{\prime },v_{y}^{\prime },v_{z}^{\prime }\right)=vcos\theta +\left(u\times v\right)sin\theta +u\left(u\cdot v\right)\left(1-cos\theta \right)$$

with v=(1,a,0) and $u=\left(\frac{a}{\sqrt{1+a^{2}}},-\frac{1}{\sqrt{1+a^{2}}},0\right)$.

Point $S_{n}$ is given a height value $z_{S_{n}}$ by the average height of subsets $G_{1}$ and $G_{2}$. Combining point $S_{n}$ and vector $v^{\prime }$, the final cross-sectional plane $l^{\prime \prime }$ is represented by:

(9) $$v_{x}^{\prime }\left(x-x_{S_{n}}\right)+v_{y}^{\prime }\left(y-y_{S_{n}}\right)+v_{z}^{\prime }\left(z-z_{S_{n}}\right)=0$$

The final cross-section is extracted using the projection of the nearby points onto plane $l^{\prime \prime }$, where the nearby points whose orthogonal distances to plane $l^{\prime \prime }$ are less than $d^{\prime }/2$ are extracted from the raw tunnel point clouds.

#### 2.2.2. A Filtering Algorithm for Non-Lining Points Removal

Many morphological filtering methods that are frequently employed in signal processing, image analysis, and bare-earth extraction fields [29,30,31], are applicable for noise removal. It is difficult to find a simple function to approximate a non-circular lining cross-section, especially when a tunnel lining has deformed. Similar to the idea of the morphological erosion, we propose an angle-based filter for removing non-lining points without the limit of the shape of tunnel lining cross-section.

**Figure 7:**
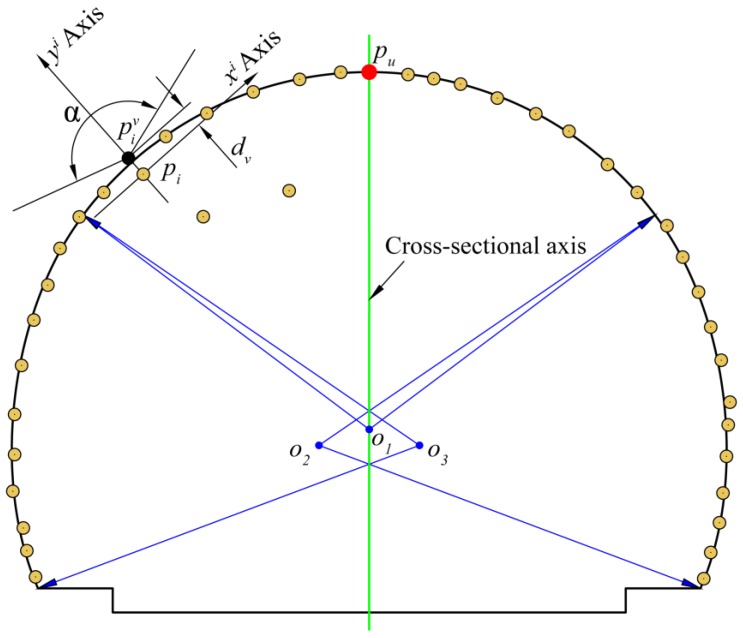
Elements of the designed filter.

As shown in Figure 7, the theoretical cross-section of the tunnel consists of three tangent circles, and it can be absolutely positioned onto the final cross-sectional plane $l^{\prime \prime }$ by using the central axis (the intersection of plane $l^{\prime \prime }$ and the vertical plane traversing centerline $l_{c}$) of the extracted cross-section and vertex $p_{u}$ (the intersection of plane $l^{\prime \prime }$ and line $l_{u}$ are illustrated in Figure 6). After the location of the theoretical cross-section, the operator of our filter is defined as an angle of α degrees (α<180°). The vertex of the angle is positioned at each cross-sectional point $p_{i}$, and the two sides of the angle make an angle α/2 with the positive $y^{i}$ axis, where the $y^{i}$ axis is taken to be along the normal line to the theoretical cross-section at point $p_{i}$, and the $x^{i}$ axis is drawn through $p_{i}$ perpendicular to the $y^{i}$ axis. The designed filter searches for cross-sectional points inside it. Since the surface of the tunnel lining is normally rough, we add a confidence interval $d_{v}$ (point $p_{i}$ is shifted a distance of $d_{v}$ along the positive $y^{i}$ axis to be point $p_{i}^{v}$) for our filter. A point $p_{i}$ ($p_{i}^{v}$) at the vertex of the angle is accepted as a lining point if there are no other cross-sectional points $p_{j}$ inside the angle. The filter function for defining the set Lp of lining points is mathematically represented as follows:

(10) $$Lp=\left\{p_{i}\in CSp|\forall p_{j}\in CSp:|x_{p_{j}}^{i}|tan\frac{\alpha }{2}+d_{v}>y_{p_{j}}^{i}\right\}$$

where CSp is the set of all cross-sectional points.

## 3. Experimental Result and Discussion

### 3.1. Data Acquisition

**Figure 8:**
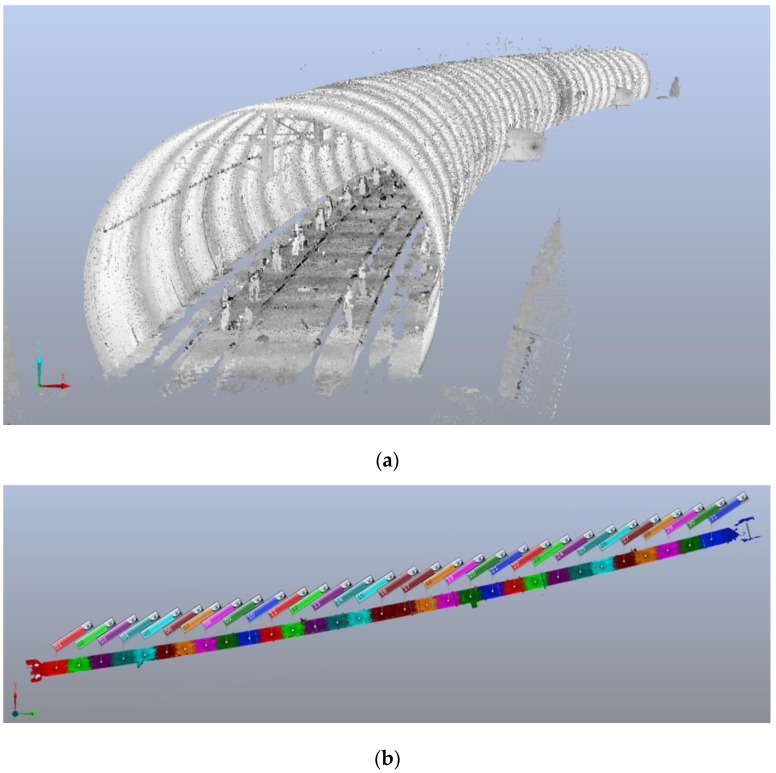
The experimental dataset. (**a**) Tunnel point clouds; (**b**) the result of the scanning position registration, where the point cloud data of each scan is represented using a different color.

The proposed method was tested in a double-track railway tunnel with the length of 619 m in Sichuan, China. As shown in Figure 8, the point cloud dataset was captured by the Faro X130 terrestrial laser scanner (Lake Mary, FL, USA) with 31 scans, and the distance of adjacent scans was about 20 m. Three sphere reference targets were laid between the two adjacent scans, which ensures the stability of the scanning position registration. All scans were registered together in a user-specified coordinate system using Faro Scene software (Lake Mary, FL, USA). The details of the point cloud dataset are listed in Table 1. MATLAB (Natick, MA, USA) was used to implement the data processing and analysis, as well as the visual representation in the following subsections.

### 3.2. Detection of Tunnel Boundary Points in the X-Y Plane

**Figure 9:**
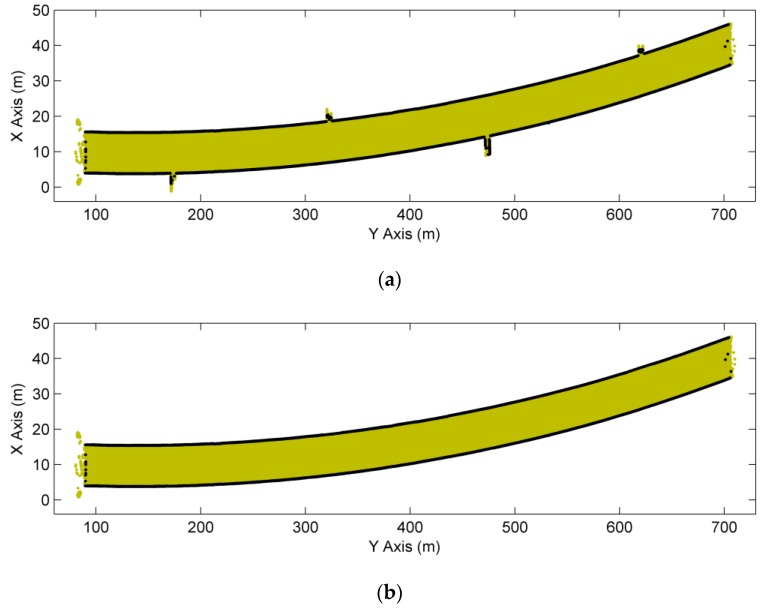
Extraction of boundary points. (**a**) Existence of noise; and (**b**) a better extraction.

As described in Section 2.1, the tunnel point cloud dataset was projected onto the X-Y plane and then discretized to improve the speed of extraction using a 0.5 m resolution grid (the width of this tunnel is about 10 m). As shown in Figure 9a, the dark dots are the extracted boundary points, but some of them are the boundary points of refuge recesses and will affect the determination of the final cross-sectional planes. The polynomial fitting was used for smoothing of tunnel boundary lines and the elimination of outliers, and the RMSE of the discrepancies is 46.5 mm. It is possible to eliminate these points of refuge recesses for a better extraction of tunnel boundary points (Figure 9b), because the heights of them are lower than the nearby lining-boundary points and the projections of them in the X-Y plane are outside the fitted boundary lines.

### 3.3. Extraction of Cross-Sections

#### 3.3.1. Extracting Results

**Figure 10:**
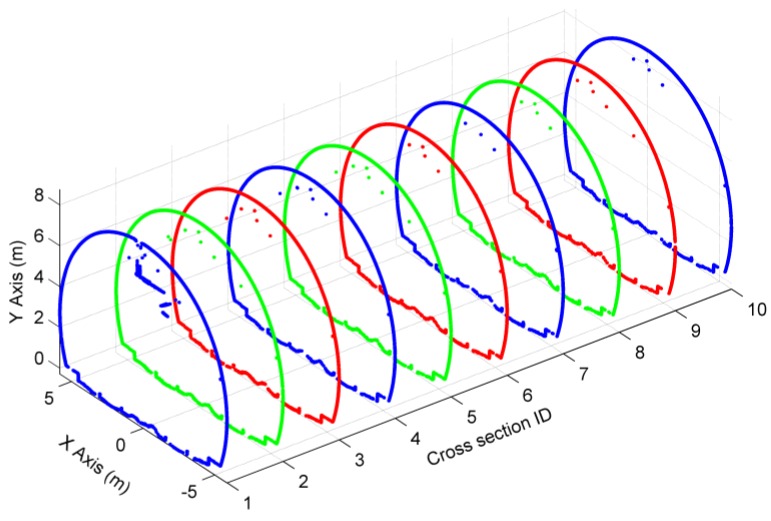
Extraction of ten cross-sections.

One of the two fitted boundary lines was used to determine the initial cross-sectional planes. Since the discrepancies of the fitted boundary line are slightly large, the initial directions are inaccurate. To optimize the directions of cross-sectional planes, the proposed method in Section 2.2.1 was implemented to obtain the final directions by using d = 40 cm. Based on the final cross-sectional planes, and by using $d^{\prime }$ = 5 mm, ten cross-sections were extracted to test the accuracy of our method and shown in Figure 10.

#### 3.3.2. Assessment of Extracting Accuracy

**Figure 11:**
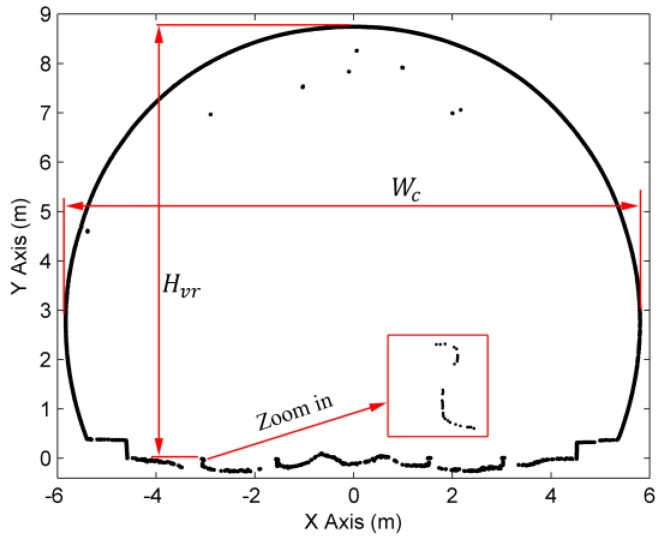
The two measures used for comparison with Han’s method.

The proposed method was compared to the method of cross-sectional estimation as specified by Han et al. [12] since Han’s method has achieved a high accuracy in comparison to total station surveying. As shown in Figure 11, two comparisons were made for the cross-sections extracted from the same location: (1) the clear width $W_{c}$ of the cross-section; and (2) the height $H_{vr}$ from the vertex of cross-section to the top of the inner rail. As shown in Figure 6, the theoretical discrepancy (Δh) between the height (h) of our cross-section and the height ($h^{\prime }$) of Han’s (cross-sections are extracted using a vertical plane) can be represented as follows:

(11) $$\{\begin{array}{c} \Delta h=h-h^{\prime }\\ h^{\prime }=\sqrt{h^{2}+{\left(h\cdot tan\theta \right)}^{2}} \end{array}$$

Let h = 8.7 m (the theoretical value of $H_{vr}$) and tanθ = 0.021 (ten cross-sections located where the theoretical tunnel grade is 21‰) then Δh = −1.9 mm. The average discrepancies of $W_{c}$ and $H_{vr}$ are −0.4 mm and −2 mm, respectively, and the RMSEs are 0.8 mm and 2.1 mm (Table 2). The clear widths of cross-sections extracted by the proposed method are very close to Han’s, and the height discrepancies are very close to the theoretical discrepancy (Δh). Specifically, the height discrepancy will increase with an increase in tunnel grade. The discussions above indicates that our method is able to offer high accuracy and more reliable tunnel cross-sections. In another word, the horizontal coordinates of cross-sectional points achieve high accuracy, and the vertical coordinates are more reliable.

### 3.4. Removing Non-Lining Points Using the Proposed Filter

#### 3.4.1. Parameter Selection and Performance Assessment

**Figure 12:**
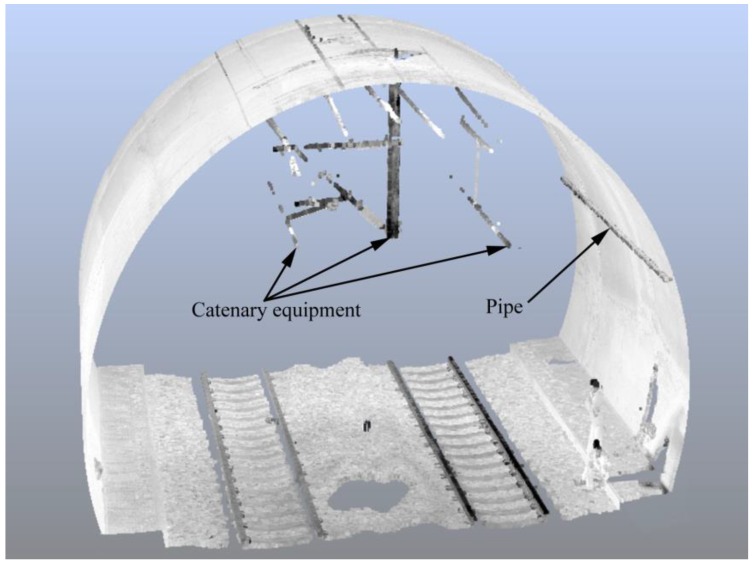
Pipes and equipment attached to the lining.

**Figure 13:**
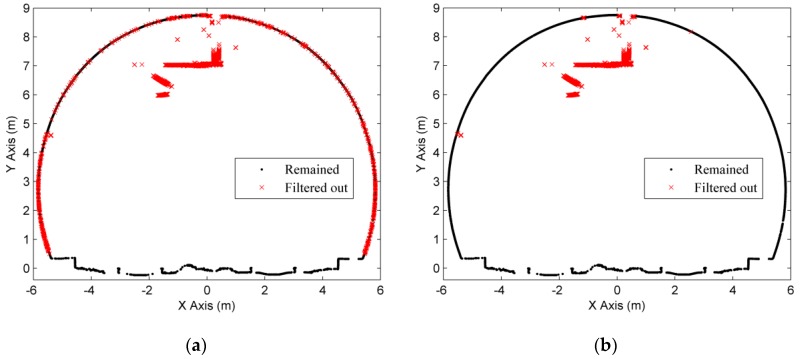
Determination of the confidence interval $d_{v}$. (**a**) $d_{v}$ = 0; and (**b**) $d_{v}$ = 1 cm.

Figure 10 shows that there are a lot of non-lining points in the cross-sections. These non-lining points belong to pipes and catenary equipment (Figure 12). To eliminate non-lining points’ interference in the safety assessment of tunnel linings, the filtering algorithm mentioned in Section 2.2.2 was used for the removal of non-lining points. The angle α of the operator must be large enough to ensure that almost all non-lining points are removed while, at the same time, the confidence interval $d_{v}$ cannot be set to a value that is too small; otherwise the operator will lose a lot of real lining points. The example in Figure 13a shows that 29.3% of lining points were removed from the cross-section (ID = 1) by using α = 165° and $d_{v}$ = 0. Hence, the confidence interval $d_{v}$ was set to 1 cm, which is slightly larger than the undulation of this tunnel surface, and only 0.275% of lining points were removed (Figure 13b).

**Figure 14:**
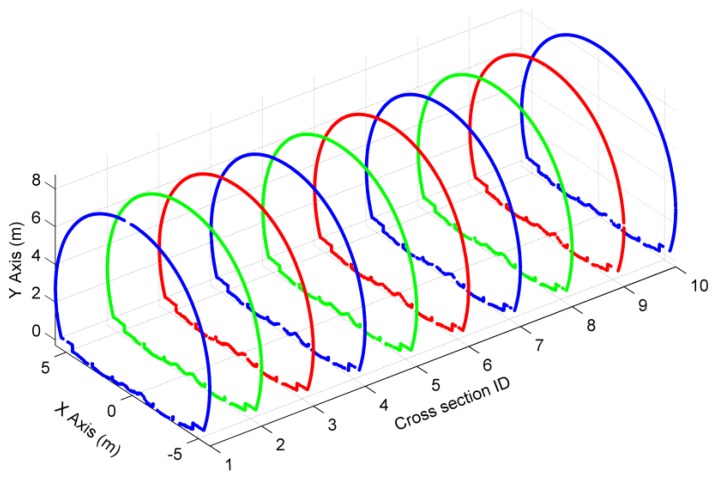
The filtering result of the ten cross-sections.

To assess the performance of the angle-based filter with different angles, the quantitative evaluations of the ten cross-sections resulted in Table 3 with counts of Type I (rejection of lining points) and Type II (acceptance of non-lining points) errors. With the increase in the angle α, Type I errors will increase; conversely, Type II errors will decrease. The angle α should be chosen to remove as many non-lining points as possible, because the lining points are sufficient to represent a complete cross-section. Finally, the angle α was set to 165°, depending on the desired proportions of Type I and Type II errors. As shown in Figure 14, the ten cross-sections extracted in Section 3.3 were filtered by using $d_{v}$ = 1 cm and α = 165°, resulting in a successful generation of ten bare-lining cross-sections. Hence, we suggest using α = 165° as the default value of the filter angle and selecting the confidence interval $d_{v}$ according to the undulation of the scanned tunnel surface.

#### 3.4.2. The Limitation of the Proposed Filter

**Figure 15:**
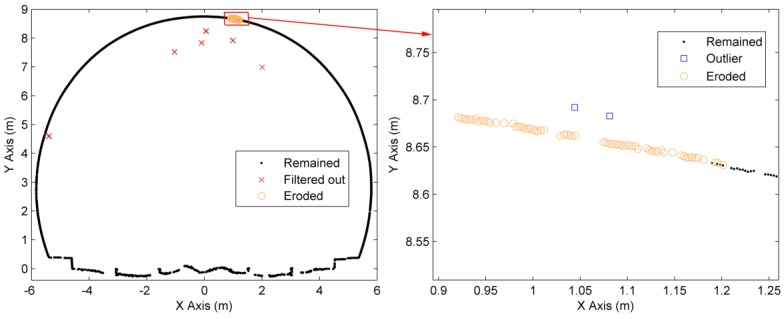
The limitation of the proposed filter.

The cross-sectional points may contain the outliers caused by multi-path reflection in TLS measurements. The proposed filter makes an assumption that the outermost points in the cross-sectional points must be classified as lining points. However, if the outliers are the outermost points, this assumption will fail. An example (ID = 3) is shown in Figure 15: the outliers eroded the lining points in their neighborhood, which produces the holes in the cross-section and impacts the performance of the filter (an increase in Type I errors).

## 4. Conclusions

We presented an automated and effective method for extracting tunnel lining cross-sections from terrestrial laser scanning (TLS) point clouds. This can be applicable in a tunnel with an arbitrarily-shaped lining cross-section. In our method, the tunnel point cloud dataset was projected onto the X-Y plane to extract the boundary points of both sides. By using these tunnel boundary points, the initial direction of the cross-sectional plane was determined, and then adjusted with the total least squares method. The cross-sectional plane, using Rodrigues’ rotation formula, was adjusted again for capturing the final cross-sectional points. To generate the bare-lining cross-section, an angle-based filter algorithm was developed for removing non-lining points based on the morphological erosion.

The proposed method was validated on the point cloud dataset of a real railway tunnel. The results of cross-sectional extraction were compared with an existing method, which showed that the clear widths of cross-sections achieved high accuracy (RMSE of 0.8 mm) and the cross-sectional heights were more reliable. The results of no-lining point removal indicated that the proposed filter was able to offer a good classification for cross-sectional points. The performance of the filter will deteriorate with the outermost outliers of the tunnel point clouds increasing; how to reduce these outliers is among our planned future work.

## Figures and Tables

**Table 1:** Details of the TLS scanning data.

Categories	Specifications
Instrument	Faro X130
Scan Angular Resolution	0.036°
Beam divergence	0.19 mrad
Range	0.6 m~130 m
Ranging error	± 2 mm
Number of points	1371 million

**Table 2:** Comparison of cross-sectional extraction accuracies.

ID	$W_{c}$(m)				$H_{vr}$(m)		
	Proposed Method	Han’s Method	Discrepancy		Proposed Method	Han’s Method	Discrepancy
1	11.6346	11.6339	0.0007		8.7365	8.7386	−0.0021
2	11.6419	11.6432	−0.0013		8.7377	8.7395	−0.0018
3	11.6332	11.6340	−0.0008		8.7378	8.7398	−0.0020
4	11.6188	11.6184	0.0004		8.7394	8.7409	−0.0015
5	11.6211	11.6209	0.0002		8.7416	8.7429	−0.0013
6	11.6238	11.6247	−0.0009		8.7319	8.7337	−0.0018
7	11.6267	11.6276	−0.0009		8.7313	8.7328	−0.0015
8	11.6256	11.6261	−0.0005		8.7322	8.7332	−0.0010
9	11.6283	11.6278	0.0005		8.7311	8.7338	−0.0027
10	11.6295	11.6305	−0.0010		8.7315	8.7353	−0.0038
Average			−0.0004				−0.0020
RMSE			0.0008				0.0021

**Table 3:** Performance assessment—counts of type I and type II errors ($d_{v}$ = 1 cm).

ID	Number of Points			% Error (α = 55°)			% Error (α = 110°)			% Error (α = 165°)	
	Lining	Non-Lining		Type I	Type II		Type I	Type II		Type I	Type II
1	5085	864		0.000	3.588		0.059	3.241		0.275	0.116
2	4274	30		0.000	10.000		0.000	6.667		0.023	0.000
3	3953	17		0.101	5.882		0.228	5.882		1.644	0.000
4	4225	16		0.047	18.750		0.095	6.250		0.710	0.000
5	3948	21		0.101	4.762		0.304	0.000		1.773	0.000
6	3825	15		0.026	6.667		0.052	0.000		0.444	0.000
7	3421	25		0.000	8.000		0.000	4.000		0.000	0.000
8	3110	14		0.000	7.143		0.000	0.000		0.289	0.000
9	3188	17		0.063	5.882		0.125	5.882		0.878	0.000
10	3008	14		0.000	7.143		0.000	0.000		0.000	0.000
**Average**				0.034	7.782		0.086	3.192		0.604	0.012

Note: non-lining points herein do not include outliers.
